# The relationship between patients’ perceptions of care quality and three factors: nursing staff job satisfaction, organizational characteristics and patient age

**DOI:** 10.1186/1472-6963-14-466

**Published:** 2014-10-18

**Authors:** Tarja Kvist, Ari Voutilainen, Raija Mäntynen, Katri Vehviläinen-Julkunen

**Affiliations:** Department of Nursing Science, University of Eastern Finland, P.O. BOX 1627, FI-70211 Kuopio, Finland; Kuopio University Hospital, P.O. BOX 1777, FI-70211 Kuopio, Finland

**Keywords:** Quality of care, Patients, Job satisfaction, Nursing staff, Hospital unit, Survey

## Abstract

**Background:**

The relationship between nurses’ job satisfaction and their perceptions of quality of care has been examined in previous studies. There is little evidence, however, about relationships between the job satisfaction of nursing staff and quality of care perceived by the patients. The aim of this study was to analyze, how the job satisfaction of nursing staff, organizational characteristics (hospital and unit type), and patients’ age relate to patients’ perceptions of the quality of care.

**Methods:**

The study was cross-sectional and descriptive, based on a secondary analysis of survey data acquired during the *At Safe* study in Finland. The study included 98 units at four acute care hospitals between autumn 2008 and spring 2009. The participants were 1909 patients and 929 nursing staff. Patients’ perceptions of quality of care were measured using the 42-item RHCS questionnaire. Job satisfaction of nursing staff was measured with the 37-item KUHJSS scale. Statistical analyses included descriptive statistics, principal component analysis, *t*-tests, analysis of variance, linear regression, and multivariate analysis of variance.

**Results:**

Patients’ perceptions of overall quality of care were positively related to general job satisfaction of nursing staff. Adequate numbers of staff appeared to be the clearest aspect affecting quality of care. Older patients were more satisfied with staff number than younger patients. Patients cared for in outpatient departments felt more respected than patients in wards, whereas patients in wards reported better care of basic needs (e.g., hygiene, food) than outpatients.

**Conclusions:**

The evaluation of resources by nursing staff is related to patients’ perceptions of the adequacy of nursing staff levels in the unit. The results emphasize the importance of considering patients’ perceptions of the quality of care and assessments by nurses of their job satisfaction at the hospital unit level when evaluating quality of care.

## Background

Previous studies have shown that the job satisfaction of nursing staff is related to their evaluation of the quality of care provided in their hospital unit [1–8]. Job satisfaction can be defined as the extent to which employees like their jobs [9–11]. It is an emotional state that is enhanced by achieving desired results at work [12] and the feeling of belonging to an efficiently functioning work community [13–16]. Job satisfaction is dependent on several factors, including the patient-to-staff ratio, quality of the working environment, nurses’ professional autonomy, respect for nurses, relationships between staff members and leaders, commitment to the organization and the amount of stress experienced [17–20]. One of the most significant factors that influences job satisfaction and nurses’ evaluations of the quality of care provided at the unit level is the practice environment and the availability of adequate resources [21–23]. If nurses are unable to carry out nursing interventions to an appropriate standard, they will not be satisfied with their jobs [4, 24, 25]. This suggests that the quality of care can be improved by adjusting factors that affect job satisfaction [26–28].

While several recent studies [5, 22, 23] have described the relationship between nurses’ job satisfaction and their perceptions of the practice environment and quality of care, it would be interesting and useful to examine the relationship between nurses’ evaluations of their job satisfaction in relation to patients’ perceptions of the quality of their care. Previously, Finnish studies [1, 2, 18] revealed that patients were highly satisfied with their care in general. The factors “values” and “work” significantly explained the nurse-perceived quality of care [1] and the proportion of RNs, patient-to-RN ratio and RNs’ working experience were highly correlated with patient satisfaction [2]. Patients’ perceptions of quality of care have been found to be significantly different in different hospitals [18] and older patients consider quality of care to be higher than do younger patients. It is important to study patients’ perceptions of quality of care because they can help to improve quality [29], which tends to be higher in well organized hospitals with a low patient-to-nurse ratio [5, 8]. Recently, Boev [30] showed that the nurse managers’ roles as a part of the work environment were significantly related to patient satisfaction.

The aims of this study were to analyze:

1. the relationship between the job satisfaction of nursing staff and patients’ perceptions of the quality of care
2. the relationship between hospital and unit type and patients’ perceptions of the quality of care
3. the relationship between patients’ age and their perceptions of the quality of care.

## Methods

### Design and sample

This study was based on a secondary analysis of data acquired during the *At Safe* study between autumn 2008 and spring 2009 [31]. The study used a cross-sectional, descriptive design and was conducted in four Finnish hospitals – one university hospital and three specialized central hospitals. The number of beds in these hospitals ranges from 377 to 784, with an average unit size of 30 beds. Data for the patient survey were collected from patients who had received care in September 2008 (hospitals A, B and C) or December 2008 (hospital D). The questionnaires were posted to the patients (*n* = 7139) at their home addresses in November 2008 (hospitals A, B and C) or February 2009 (hospital D). The patients were randomly chosen within the hospital units so that approximately 10% of patients cared for in each unit in a month received the questionnaire. A total of 2566 out of the 7139 patients contacted (36%) responded to the questionnaire. No reminders were sent. Participation in the study was voluntary and anonymous.

Data relating to the job satisfaction of nursing staff were collected with the assistance of nursing leaders in each of the hospitals studied. The researchers made contact with the leaders to explain the purpose of the study and how data should be collected. The nursing leaders informed the members of the nursing staff about the study and encouraged them to fill in the questionnaire. Basic information about the study was also provided on each hospital’s internal website. An electronic version of the questionnaire was sent by e-mail to each member of the nursing staff at three hospitals (*n* = 3708), and a reminder e-mail was sent if they failed to return the questionnaire within three weeks. In one hospital (*n* = 2070), a paper version of the questionnaire was posted to the home addresses of nursing staff in October 2008 and no reminders were sent. A total of 2708 of the 5778 nursing staff (47%) responded. Participation in the study was voluntary and anonymous.

As we wanted to associate the results from the job satisfaction survey to those from the quality of care survey, we were forced to pool the data using the lowest common denominator linking the two surveys, i.e. the hospital unit. The patients evaluated their perceptions of the unit as a whole, rather than individual members of staff, which made it impossible to relate quality of care to job satisfaction at the individual level. Consequently, we chose to compare average job satisfaction ratings to the average quality of care at the unit level. Units where fewer than six patients and/or fewer than three staff members responded to the questionnaires were systematically excluded from the secondary dataset analyzed, yielding a dataset comprising responses from 1909 patients and 929 staff members.

The number of hospital units included in this secondary analysis was 98 (56 wards and 42 outpatient departments). Of these units, 34 were in hospital A (the university hospital), 15 in central hospital B, 22 in central hospital C and 27 in central hospital D. All four hospitals provide specialized care, but the central hospitals do not have as many specialties as the university hospital. The dataset included responses from units representing all of the common medical specialties. However, due to the low number of responses from patients in psychiatric units, the final dataset did not include any information relating to psychiatric wards and only three sets of responses for psychiatric outpatient departments (Table 1).

**Table 1:** **Type and number of the units in hospitals**

Type of unit	Hospital A	Hospital B	Hospital C	Hospital D
**Wards**				
Medical	5	1	6	4
Surgical	5	3	5	5
Maternity and gynecology	4	1	2	1
Eye diseases	1			
Ear diseases	1		1	1
Dermatology	1			
Cancer	1			
Neurology	1	2	1	1
Pulmonary diseases	1	1		1
Rehabilitation				1
**Outpatient departments**				
Surgical	1	1	1	1
Medical	1	1	1	1
Maternity and gynecology	2			2
Eye diseases	1	1	1	1
Ear or tooth and mouth diseases	3		1	2
Dermatology	1	1	1	1
Cancer	1			2
Pulmonary diseases	1			1
Rehabilitation	1	1		1
Psychiatry	1	1	1	
Emergency	1	1	1	
Dialysis				1
**Total**	34	15	22	27

We also examined hospital (A-D) and unit type (ward or outpatient department) in relation to patients’ perceptions of the quality of care in addition to the job satisfaction of nursing staff. In the analysis, hospital and unit type were treated as fixed factors (categorical variables), which allowed us to examine possible differences in perceptions of the quality of care between the patients in different hospitals and/or unit types. Furthermore, patients’ age may affect their perceptions [2, 32] and this was included as a statistical covariate (continuous variable) in the analysis.

### Instruments

Patients’ perceptions of quality of care were measured using the Revised Humane Caring Scale (RHCS). The original Humane Caring Scale (HCS) was developed in Kuopio University Hospital in the early 1990s and has been used to measure the quality of care as evaluated by patients [1, 2]. The revised version (RHCS) was shown to be practical in a contemporary context in a pilot study in 2007 [18, 33] and consists of 46 Likert-scaled items relating to quality of care, with possible responses ranging from 1 = *strongly disagree* to 5 = *strongly agree*. Four of the 46 items are less specific than the other items and provide a measure of the overall quality of care rather than specific aspects (Table 2). These four general items were excluded from the analyses in this work as they could have complicated the interpretation of the results of the multivariate analyses.

**Table 2:** **Principal component analysis of patient data (** ***n*** **= 1909) concerning quality of care**

Component’s name and summary				Statements included in the components
1. Mutual respect	0.93^b^	8.8^c^	21^d^	I was appreciated (0.78^e^)
4.44, 3.73-4.97^a^				I was able to speak with the staff in private (0.78)
				I felt welcomed into the hospital (0.76)
				I felt safe in hospital (0.74)
				I was able to discuss issues with the staff in confidence (0.70)
				I was listened to when I had worries (0.67)
2. Information	0.95	7.9	19	Restrictions relating to my illness were explained to me (0.78)
4.24, 3.56-4.75				I was given clear instructions about home care (0.77)
				I received sufficient information about my home care (0.76)
				I received sufficient information about my illness (0.76)
				I received sufficient information about my medication (0.68)
				The rules relating to the hospital environment were explained (0.64)
				I was able to ask questions concerning my care (0.61)
				I was able to participate in the planning of my care (0.61)
				I was addressed in clear and intelligible language (0.56)
				The staff relied on my own assessment of how I felt (0.54)
				My family were given enough attention (0.50)
				Sufficient concern was shown about my state of health (0.49)
				The members of staff respected each other’s expertise (0.48)
3. Basic needs	0.93	5.2	12	I was helped with my personal hygiene if necessary (0.94)
4.27, 3.42-4.97				I was given enough to drink (0.94)
				I was given an appropriate amount of food (0.93)
				I was able to maintain and/or improve my mobility (0.86)
				I received medication for my pain at the right time (0.59)
				My pain was noticed and taken seriously (0.49)
4. Expertise	0.94	4.3	10	The physicians were professional (0.71)
4.52, 3.89-4.93				The other staff were professional (0.69)
				The nursing staff were professional (0.60)
				I received help when I needed it (0.59)
				There was good collaboration between members of staff (0.44)
				I was treated in a friendly way (0.40)
				I was treated with respect (0.40)
				I was accepted for what I was (0.39)
				My fears were alleviated (0.36)
				My treatment was based on my needs (0.32)
5. Staffing adequacy	0.92	3.2	7	There were enough members of staff (0.74)
4.04, 3.36-4.80				The atmosphere was unhurried (0.69)
				The staff had enough time for me (0.67)
				The atmosphere was positive (0.50)
				The staff showed just the right level of interest (0.40)
6. Pain relief	0.69	2.1	5	I was given understandable guidance about pain treatment (0.66)
3.62, 2.20-4.60				My pain was also relieved with non-medical treatments (0.62)

^a^Mean, minimum-maximum score at the hospital unit level.

^b^Cronbach’s α.

^c^Eigenvalue (λ).

^d^% of the original variability explained by the component.

^e^Component loading.

Job satisfaction of nursing staff was measured using the Kuopio University Hospital Job Satisfaction Scale (KUHJSS) [18, 19], which was developed as part of this research project. The initial version of this scale was drawn up on the basis of a literature review and it was then revised and amended on the results of pilot studies and feedback from expert panels. The KUHJSS includes 37 Likert-scaled items (Table 3), with possible responses ranging from 1 = *strongly disagree* to 5 = *strongly agree.* All 37 items were considered in the analysis presented herein [19].

**Table 3:** **Principal component analysis of nursing staff data (** ***n*** **= 929) concerning job satisfaction**

Component’s name and summary				Statements included in the components
1. Leadership	0.94^b^	5.8^c^	16^d^	My manager is interested in staff well-being (0.91^e^)
3.83, 2.57-4.63^a^				…provides the staff feedback with an aim to develop work (0.88)
				…encourages the staff to take part in the planning (0.88)
				…treats the staff fairly and equally (0.86)
				…informs well about issues concerning my unit (0.79)
				…is interested in work results and outcomes (0.78)
				…enables continuous professional development (0.73)
2. Staff resources	0.85	3.3	9	New employees are familiarized well in my unit (0.76)
3.20, 2.12-4.30				The workload is distributed evenly in my unit (0.67)
				The flow of information works well in my unit (0.63)
				There is usually enough staff in my unit (0.61)
				My workload is appropriate (0.54)
3. Working preconditions	0.77	3.2	9	My salary is appropriate in relation to my work (0.79)
3.32, 2.28-4.30				I am satisfied with my working hours (0.64)
				I do not find my work too stressful (0.56)
				I am willing to work in the hospital district in the future (0.55)
4. Working conditions	0.85	3.2	9	My unit has appropriate work facilities (0.85)
3.41, 2.13-4.60				My unit is comfortable (0.84)
				My unit is safe and secure (0.49)
				My unit has equipment to ensure quality of care (0.80)
5. Self-Appreciation	0.79	3.1	8	I look after my own personal well-being (0.83)
4.09, 3.22-4.78				I am happy with my current health (0.69)
				I am active in developing myself professionally (0.66)
				I feel I am a competent employee (0.61)
				Combining work and personal life is successful (0.59)
6. Independence	0.80	2.4	7	I have the opportunity to make independent decisions (0.79)
4.13, 3.30-4.83				I have the opportunity to plan my work independently (0.62)
				I have a chance to influence decision-making in my unit (0.38)
7. Professional self-esteem	0.74	2.4	6	I appreciate my own work (0.76)
				My work is interesting (0.59)
4.52, 3.60-5.00				Client feedback motivates me in my work (0.57)
				I trust the expertise of my colleagues (0.48)
8. Balance between skills and tasks	0.69	2.4	6	I can apply my skills and expertise in my work (0.76)
3.99, 3.00-4.83				My work tasks are suitably challenging (0.65)
				There is a good community spirit in my unit (0.65)
9. Ambitions	0.64	1.5	4	I have a chance for career development (0.80)
2.70, 1.40-3.70				The upper management appreciates my work (0.46)

^a^Mean, minimum-maximum score at the hospital unit level.

^b^Cronbach’s α.

^c^Eigenvalue (λ).

^d^% of the original variability explained by the component.

^e^Component loading.

### Ethical considerations

The study design was reviewed and approved by the Research Ethical Committee of the Northern Savo Hospital District (Permission number 46/2007). In addition, research permission was given by the chief executive medical directors, chief nursing officers and personnel managers of all four hospitals. In each case the survey documents and questionnaires included the researchers’ contact details and information about the study. Participation was voluntary and anonymous.

### Data analyses

Data were analyzed using IBM SPSS 19.0 for Windows (Chicago, Illinois). The categorical variables were examined using frequencies, percentages and ranges. The distributions and central tendencies of continuous demographic variables were evaluated in terms of their means and standard deviations. Student’s *t-*tests and analysis of variance (ANOVA with Tukey’s HSD post-hoc test) were used to test for statistically significant differences in the ages of patients and nursing staff and in the work experience of nursing staff by hospital and unit type. *P*-values <0.05 were considered to be statistically significant in all analyses.

To be able to evaluate results better, we provided a missing value analysis on the patient (*n* = 1909) and staff member datasets (*n* = 929) prior to pooling them over the units. In general, item nonresponse can act as a significant confounding factor in the analysis of patient surveys [34]. The present analysis revealed that the proportion of missing values was less than 5% in 34 out of 43 variables tested (42 questionnaire items and age) in the patient dataset. The proportion of missing values was highest for the items “I was able to maintain and/or improve my mobility” (19.7%), “I was helped with my personal hygiene if necessary” (19.3%), “I was given an appropriate amount of food” (17.0%), “I was given enough to drink” (16.9%) and “As well as using medicine, my pain was relieved with other treatments” (15.9%). In each of these cases, the proportion of missing values was evidently higher in outpatient departments (25 – 41%, range) than in wards (1 – 10%). Moreover, for the item listed, the average age of the patients who had not answered the question was higher than that of those who had answered it (60 *vs.* 55 years, Student’s *t*-test, *t*_440_ = -5.4, *p* <0.001). In the staff members dataset, the proportion of missing values was less than 5% for all variables tested (37 questionnaire items).

Principal component analysis (PCA) with orthogonal varimax rotation was used as the method for examining data from patient and staff questionnaires. Prior to running the PCAs, the average score of each Likert-scale question was calculated for every unit and standardized to give a mean of zero and a standard deviation (SD) of one. In the text below, the PCA performed using data from the patient questionnaire is termed the patient PCA (PPCA). Similarly, the PCA performed using data from the staff questionnaire is termed the staff PCA (SPCA). The significance of PCA loadings was determined according to the broken-stick criterion, which is described in detail by Peres-Neto *et al*. [35]. Briefly, this criterion weights the significance of a loading relative to the significances of adjacent loadings within the same row but also within the component to which the loading in question belongs. Reliability of the principal components produced was evaluated on the basis of their Cronbach’s alpha values (Tables 2 and 3). An alternative idea was to perform separate PCA for ward and outpatient department data, but this proved to be technically unsuitable as the number of outpatient departments was lower than the number of questionnaire statements which we wanted to include in the analyses.

The relationship between patients’ perceptions of overall quality of care and nurses’ general job satisfaction was first modeled by fitting a simple linear regression to the entire data set. In this case, the overall quality of care referred to the mean value of the RHCS and the general job satisfaction to the mean value of the KUHJSC per hospital unit. In addition to the simple linear regression, a two-way multivariate analysis of variance (MANOVA) was used to test the effects of job satisfaction, organizational characteristics (hospital and unit type), and patients’ age on perceptions of the quality of care. Component scores were used as dependent variables in the MANOVA. Hospital (A – D) and type of unit (ward or outpatient department) were used as fixed factors and the component scores of the SPCA together with the patients’ ages were used as covariates in the MANOVA. On the basis of preliminary analyses, we were able to exclude the staff members’ ages and the length of their working experience from the analyses, as these were not related to any of the dependent variables. This helped to meet the statistical assumptions of MANOVA.

## Results

### Respondent demographics

Responses from 1909 patients were considered in this work. Their ages ranged from 15 to 94 with a mean of 56 and SD of 17 years. In total, 1067 of the patients were female, 780 were male and 62 did not state their gender. The mean age of the patients differed across the hospitals, with the patients in hospital D (age 54 ± 16 years, mean ± SD) being younger than those cared for in hospitals B and C (58 ± 16 years, in both cases) (ANOVA and Tukey’s HSD post-hoc test, *p* <0.05). Moreover, the patients cared for in hospital A were younger on average (55 ± 17 years) than those cared for in hospital C (ANOVA and Tukey’s HSD post-hoc test, *p* <0.05). The age of the patients also differed between the wards and outpatient departments. On average, patients cared for in the wards were older (57 ± 17 years) than those cared for in the outpatient departments (54 ± 17 years; *t*-test, *p* <0.001) (Table 4).

**Table 4:** **Demographics of the patients (n = 1909)**

Background variable	%
Gender	
Female	55.9
Male	40.9
Missing	3.2
Age	
<20 years	0.9
20-29 years	8.8
30-39 years	9.3
40-49 years	12.0
50-59 years	22.5
60-69 years	22.6
70-79 years	16.7
>79 years	5.9
Missing	1.3
Living	
Alone	22.3
With a spouse	76.7
Missing	1.0
Education	
University degree	10.7
University of applied sciences degree	9.1
Vocational degree	46.4
No degree	25.8
Other	5.4
Missing	2.6
Occupational status	
Senior management/professional	6.2
Junior management/clerical	10.2
Self-employed. farmer	6.2
Employed	22.9
Pensioner	45.6
Other	7.7
Missing	1.2
Hospital admission	
Planned	67.5
An emergency	30.0
Missing	2.5
Reason for admission to hospital	
Examination	26.2
Treatment	56.5
Other	6.6
Missing	0.7

Responses from 929 nursing staff members were considered. Their ages ranged from 19 to 67 with a mean ± SD of 43 ± 10 years; 98% of them (*n* = 912) were female. On average, members of the nursing staff had 17 ± 11 years (mean ± SD) of work experience. The mean age of the respondents did not differ across the hospitals (ANOVA and Tukey’s HSD post-hoc test, *p* >0.05) but, on average, staff at hospital C had less work experience (15 ± 10 years, mean ± SD) than staff at hospitals A and D (18 ± 11 years, in both cases) (ANOVA and Tukey’s HSD post-hoc test, *p* <0.05). The ages and work experience of the staff did not differ between the wards and outpatient departments (*t*-test, *p* >0.05 in both cases) (Table 5).

**Table 5:** **Demographics of nursing staff (n = 929) (%)**

Background variable	%
Gender	
Female	94.0
Male	4.2
Missing	1.8
Age	
<20 years	0.3
20-29 years	14.0
30-39 years	23.4
40-49 years	31.2
50-59 years	26.7
>59 years	1.9
Missing	2.7
Profession	
Nurse leader	6.1
Nurse, midwife, public health nurse, physiotherapist, radiographer, lab nurse	74.7
Practical nurse	15.7
Other	2.5
Missing	1.0
Type of employment	
Permanent	79.3
Temporary	19.6
Missing	1.1
Working hours	
Day	30.4
Rotational	68.5
Missing	1.1

### Principal component analyses of patient and nursing staff survey data

The correlation matrix derived from the patient survey results was suitable for PCA (Kaiser-Meyer-Olkin: Measure = 0.89, Bartlett’s Test: *p* <0.001) and yielded six principal components with eigenvalues (λ) >1 (Table 2). The correlation matrix derived from data from the staff questionnaire was also suitable for PCA (Kaiser-Meyer-Olkin: Measure = 0.82, Bartlett’s Test: *p* <0.001), and yielded nine principal components having eigenvalues (λ) >1 (Table 3).

### Relationships between quality of care and explanatory variables

**Figure 1:**
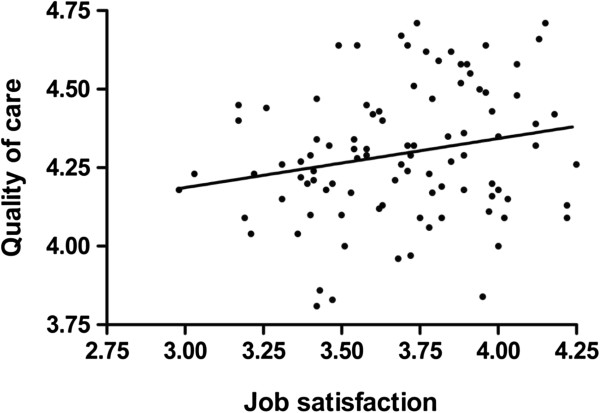
**Relationship between patients’ perceptions of overall quality of care and nurses’ general job satisfaction.** The overall quality of care refers to the mean value from the patient questionnaire; general job satisfaction refers to the mean value from the staff questionnaire per hospital unit (*n* = 98, indicated with black dots). The solid line represents a linear regression.

The simple linear regression with overall quality of care as the dependent variable and general job satisfaction as the independent variable was statistically significant (ANOVA, *F*_1, 96_ = 4.63, Pearson’s correlation coefficient *r* = 0.21, *p* = 0.034), but explained only 5% of the original variation in the overall care quality (coefficient of determination *r*^2^ = 0.046). According to the model, when general job satisfaction increased by 1, the overall quality of care increased by 0.16 (*y* = 3.719 + 0.156*x*, Figure 1). One outlier with a standardized residual of -2.93 in the primary model was removed from the data prior to building the final model. In the final model, standardized residuals were normally distributed (Kolmogorov-Smirnov test, *p* = 0.856) and their absolute values were all <2.5.

**Figure 2:**
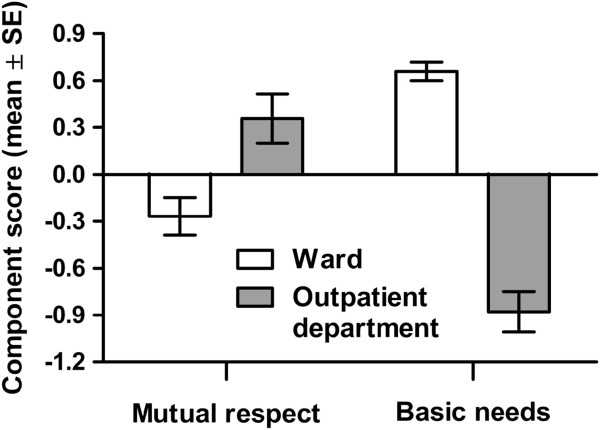
**Effect of unit type (ward or outpatient department) on patients’ perceptions of mutual respect (patient survey component 1) and fulfilling the basic needs (patient survey component 3).**

**Figure 3:**
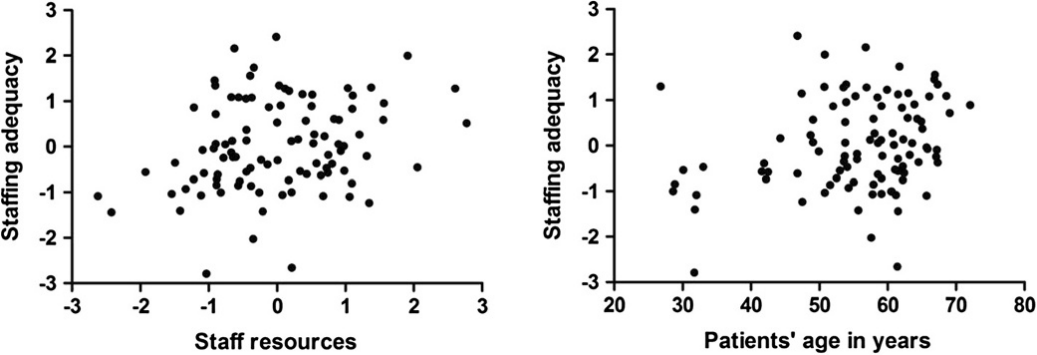
**Relationships between patients’ perceptions of staffing adequacy (patient survey component 5) and nurses’ evaluations of staff resources (staff survey component 2, on the left) and patients’ ages (on the right).**

All relationships between the six components of patients’ perceptions of quality of care and explanatory variables (nine components of nurses’ job satisfaction, four hospitals, two unit types, and patients’ age) were studied. For the sake of clarity, only the statistically significant relationships are presented in the following paragraphs as well as Figures 2 and 3. Multivariate tests using MANOVA indicated that the PPCA components were related to the unit in charge of the patient’s care (Wilks’ λ: *F* = 20.66, *r*^2^ = 0.60, *p* <0.001), SPCA component two (*F* = 2.54, *r*^2^ = 0.16, *p* = 0.035), and the mean age of the patients cared for in that unit (*F* = 3.34, *r*^2^ = 0.19, *p* = 0.009). All dependent and independent variables used were distributed normally (Kolmogorov-Smirnov: *p* >0.05 in each case), thus justifying the use of MANOVA.

Based on tests of between-subjects effects, the first PPCA component (the “mutual respect component”) exhibited a relationship to the type of unit caring for the patient (*F*_1, 17_ = 6.28, *p* = 0.014), with higher scores being observed for outpatient departments (4.52 ± 0.29, mean ± SD) than for wards (4.38 ± 0.33). The third PPCA component (the “basic needs component”) also exhibited a relationship to the type of unit caring for the patient (*F*_1, 17_ = 65.02, *p* <0.001). In this case, the component had higher scores for wards (4.53 ± 0.29) than for outpatient departments (3.92 ± 0.45). The fifth PPCA component (the “staffing adequacy component”) exhibited a relationship to the second SPCA component (the “staff resources component”) (*F*_1, 17_ = 7.50, *r* = 0.27, *p* = 0.008) and the patients’ ages (*F*_1, 17_ = 5.57, *r* = 0.25, *p* = 0.021) (Figure 1). In both cases, the relationship was linear and positive. The identity of the hospital had no effect on the PPCA components (Wilks’ λ: *F* = 1.32, *p* = 0.192).

## Discussion

In this study, responses from patients and nursing staff were combined at the unit level to examine the relationship between job satisfaction amongst nursing staff and patients’ perceptions of quality of care. This approach has seldom been used in health service and nursing research, although it has been used at the hospital level [5, 8, 30]. Patients, however, are often cared for in several units, so it might be difficult to obtain reliable evaluations of a single unit. In this study we mentioned in the information sheet that the questionnaire concerned the quality of care in the named unit, but the results still need to be interpreted with care.

Data from 98 units in four acute care hospitals in Finland were considered. Patients’ perceptions of overall quality of care were positively related to the general job satisfaction reported by nursing staff. Patients who were cared for in outpatient departments felt more respected than patients cared for on hospital wards. Meetings between patients and staff in outpatient departments are generally more private than on wards. On the other hand, patients reported that their basic needs were better met on wards than in outpatient departments. This is probably because the patients stay longer in wards than in outpatient departments and, in wards, they frequently need the help of nursing staff to meet their daily basic needs such as eating, hygiene and pain management. The proportion of missing values for the statements related to basic needs was higher for outpatient departments than for wards. This has to be taken into account when interpreting the results. We acknowledge that the results need to be verified by other studies before drawing broader conclusions. The type of hospital had no effect on patients’ perceptions of quality of care according to the current analysis, although an earlier primary analysis of the patient data did reveal differences between the patients’ perceptions of the quality of care in different hospitals. In the latter case, however, there were more data than in this secondary analysis (n = 2566) [18].

According to the results of this study, the job satisfaction of nursing staff and patients’ perceptions of quality of care appear to be related. Both low and high patient dependence on nursing care can have a positive effect on nurse job satisfaction, as reported previously [36]. According to this and previous studies [1, 2, 18, 19], Finnish nurses are very professional, highly motivated and they appreciate their work, though they are not so satisfied with the staff resources. Nurses who are satisfied with their jobs are essential for high quality care, as our results show. It may simply be the fact that nursing staff who are satisfied with their work provide high-quality patient care. A high level of job satisfaction may also be the result of a satisfactory work environment and adequate resources, which *per se* have positive effects on patients’ perceptions [8]. In the present study, patients’ perceptions of the adequacy of resources were positively linked to the nursing staff’s evaluation of their workload. Our results are supported by the findings of earlier studies using hospital-level data [5, 8, 30]. Aiken *et al.* [8] examined hospital-level data and found that good working environments and low patient-to-nurse ratios were associated with increased quality of care and patient satisfaction. In addition, Szecsenyi *et al.* [7] found that patients’ satisfaction with practice organization in primary care was positively correlated with job satisfaction among nursing staff. These results are consistent with our findings (which are based on unit-level data). These results present a challenge to nurse and hospital leaders to track and evaluate staffing ratios critically since they are important indicators of the nursing staff’s and patients’ satisfaction. Both can be managed by maintaining an appropriate number of nursing staff.

The relationship between the patients’ perceptions of the adequacy of resources and the mean age of the patients cared for in a unit was positive. One interpretation is that older patients are more satisfied with the number of staff present than younger patients. Similarly, Tervo-Heikkinen *et al.* [2] reported that older patients generated higher satisfaction sum scores including those relating to facilities. Generally, older patients are more satisfied with health services than younger ones [37].

This study found no relationship between leadership and the quality of care. This contrasts with the findings of Van Bogaert *et al.* [17] and Boev [30] but is confirmed by a previous report by Kvist *et al*. [1]. The relationship between leadership and quality of care needs multidimensional innovative research using e.g. data mining of large existing data pertaining to these topics [38].

### Limitations

We only studied the quality of care from the patients’ perspective and the job satisfaction of nursing staff at the unit level. It would be fruitful to examine the relationships between the patient to registered nurse ratio or the number of registered nurse hours per patient at the unit and the perceived quality of care. In addition, the hospitals examined in this study were all located in the same region of Finland. Although the samples were relatively large, data from a larger region would yield more general results.

## Conclusions

Generally, nursing staff with a high level of job satisfaction are important for high quality care, as evaluated by the patients. There is a positive relationship between evaluation of resources by nursing staff and patients’ perceptions of whether there are sufficient staff. This finding presents a number of challenges to nurse managers and directors who need to ensure sufficient staffing levels in their units. It is vital to support the well-being of staff because this has the potential to improve patients’ perceptions of staffing levels. Continuing professional development both increases the job satisfaction of nursing staff and ensures the presence of skilled staff, which in turn increases patient confidence in the adequacy of a unit’s staffing level. The results emphasize the importance of considering patients’ perceptions of the quality of care and assessments by nurses of their job satisfaction at hospital unit level when evaluating quality of care.

## Consent

Written informed consent was obtained from the patient for the publication of this report and any accompanying images.

## Abbreviations

KUHJSS: Kuopio University Hospital Job Satisfaction Scale
RHCS: Revised Humane Caring Scale.

## References

[1] Kvist T, Vehviläinen-Julkunen K, Jokela V (2007). Do organizational factors explain the quality of care?. J Nurs Care Qual.

[2] Tervo-Heikkinen T, Kvist T, Partanen P, Vehviläinen-Julkunen K, Aalto P (2008). Patient satisfaction as a positive nursing outcome. J Nurs Care Qual.

[3] Chang W-Y, Ma J-C, Chiu H-T, Lin K-C, Lee P-H (2009). Job satisfaction and perceptions of quality of patient care, collaboration and teamwork in acute care hospitals. J Adv Nurs.

[4] Burtson PL, Stichler JF (2010). Nursing work environment and nurse caring: relationship among motivational factors. J Adv Nurs.

[5] Purdy N, Laschinger HKS, Finegan J, Kerr M, Olivera F (2010). Effects of work environment on nurse and patient outcomes. J Nurs Manag.

[6] Hinami K, Whelan CT, Wolosin RJ, Miller JA, Wetterneck TB (2011). Worklife and satisfaction of hospitalists: toward flourishing careers. J Gen Int Med.

[7] Szecsenyi J, Goetz K, Campbell S, Broge B, Reuschenbach B, Wensig M (2011). Is the job satisfaction of primary care team members associated with patient satisfaction?. BMJ Qual Saf.

[8] Aiken LH, Sermeus W, Van den Heede K, Sloane DM, Busse R, McKee M, Bruyneel L, Rafferty AM, Griffiths P, Moreno-Casbas MT, Tishelman C, Scott A, Brzostek T, Kinnunen J, Schwendimann R, Heinen M, Zikos D, Sjetne IS, Smith HL, Kutney-Lee A (2012). Patient safety, satisfaction, and quality of hospital care: cross sectional surveys of nurses and patients in 12 countries in Europe and the United States. BMJ.

[9] Rambur B, McIntosh B, Palumbo MV, Reinier K (2005). Education as a determinant of career retention and job satisfaction among registered nurses. J Nurs Schol.

[10] Adams A, Bond S (2000). Hospital nurses’ job satisfaction, individual and organizational characteristics. J Adv Nurs.

[11] DiMeglio K, Padula C, Piatek C, Korber S, Barrett A, Ducharme M, Lucas S, Piermont N, Joyal E, DeNicola V, Corry K (2005). Group cohesion and nurse satisfaction: examination of a team building approach. J Nurs Staff Dev.

[12] Manojlovich M, Laschinger HKS (2002). The relationship empowerment and selected personality: characteristics to nursing job satisfaction. J Nurs Adm.

[13] Roberts BJ, Jones C, Lynn M (2004). Job satisfaction of new baccalaureate nurses. J Nurs Adm.

[14] Manojlovich M (2005). Linking the practice environment to nurses’ job satisfaction among nurses. J Nurs Schol.

[15] Ruggiero JS (2005). Health, work variables, and job satisfaction among nurses. J Nurs Adm.

[16] Ulrich BT, Bauerhaus PI, Donelan K, Norman L, Dittus R (2007). Magnet status and registered nurse views of the work environment and nursing as a career. J Nurs Adm.

[17] Van Bogaert P, Meulemans H, Clarke S, Vermeyen K, Van de Heyning P (2009). Hospital nurse practice environment, burnout, job outcomes and quality of care: test of a structural equation model. J Adv Nurs.

[18] Kvist T, Mäntynen R, Turunen H, Partanen P, Miettinen M, Wolf G, Vehviläinen-Julkunen K (2013). How magnetic are Finnish hospitals measured by transformational leadership and empirical quality outcomes?. J Nurs Manag.

[19] Kvist T, Mäntynen R, Partanen P, Turunen H, Miettinen M, Vehviläinen-Julkunen K: **The job satisfaction of Finnish nursing staff: the development of a job satisfaction scale and survey results.** *Nurs Res Pract* Epub 2012 Oct 23 10.1155/2012/210509 PMC3486130 23133750

[20] Lu H, Barriball KL, Zhang X, While AE (2012). Job satisfaction among hospital nurses revisited: a systematic review. Int J Nurs Stud.

[21] Van Bogaert P, Clarke S, Roelant E, Meulemans H, Van de Heyning P (2010). Impacts of unit-level nurse practice environment and burnout on nurse-reported outcomes: a multilevel modeling approach. J Clin Nurs.

[22] Hinno S, Partanen P, Vehviläinen-Julkunen K (2011). Hospital nurses’ work environment, quality of care provided and career plans. J Nurs Rev.

[23] Aiken LH, Sloane DM, Clarke S, Poghosyan L, Cho E, You L, Finlayson M, Kanai-Pak M, Aungsuroch Y (2011). Importance of work environments on hospital outcomes in nine countries. Int J Qual Health Care.

[24] Kalisch B, Arbor A, Tschanen D, Lee H (2011). Does missed nursing care predict job satisfaction?. J Healthc Manag.

[25] Randall Andrews D (2011). Nurses’ self-concept and perceived quality of care: a narrative analysis. J Nurs Care Qual.

[26] Kooker BM, Kamikawa C (2010). Succesful strategies to improve RN retention and patient outcomes in a large medical centre in Hawaii. J Clin Nurs.

[27] Brooks-Carthon JM, Kutney-Lee A, Sloane DM, Cimiotti JP, Aiken LH (2011). Quality of care and patient satisfaction in hospitals with high concentrations of black patients. J Nurs Schol.

[28] Nantsupawat A, Srisuphan W, Kunaviktikul W, Wichaikhum O-A, Aungsuroch Y, Aiken LH (2011). Impact of nurse work environment and staffing on hospital nurse outcomes and quality of care in Thailand. J Nurs Schol.

[29] Luxford K (2012). What does the patient know about quality?. Int J Qual Health Care.

[30] Boev C (2012). The relationship between nurses’ perception of work environment and patient satisfaction in adult critical care. J Nurs Schol.

[31] **Attractive and safe hospital study** http://www.uef.fi/hoitot/vetovoimainen-ja-turvallinen-sairaala-hanke

[32] Rahmqvist M (2001). Patient satisfaction relation to age, health status and other background factors: a model for comparisons of care units. Int J Qual Health Care.

[33] Kvist T, Vehviläinen-Julkunen K (2008). Ihmisläheinen hoito -mittarin kehittäminen ja innovatiivinen käyttö: (The development and the innovative use of the Humane Caring Scale). Hoitotiede (Nursing Science).

[34] Voutilainen A, Kvist T, Sherwood PR, Vehviläinen-Julkunen K (2014). A new look at patient satisfaction: learning form self-organizing maps. Nurs Res.

[35] Peres-Neto PR, Jackson DA, Somers KM (2003). Giving meaningful interpretation to ordination axes: assessing loading significance in principal component analysis. Ecology.

[36] Pitkäaho T, Ryynänen O-P, Partanen P, Vehvilainen-Julkunen K (2011). Data-based nurse staffing indicators with Bayesian networks explain nurse job satisfaction: a pilot study. J Adv Nurs.

[37] Schoenfelder T, Klewer J, Kugler J (2011). Determinants of patient satisfaction: a study among 39 hospitals in an in-patient setting in Germany. Int J Qual Health Care.

[38] Henley SJ (2014). Mother lodes and mining tools: big data for nursing science. Nurs Res.

[CR39] The pre-publication history for this paper can be accessed here:http://www.biomedcentral.com/1472-6963/14/466/prepub

